# Key Players of the Immunosuppressive Tumor Microenvironment and Emerging Therapeutic Strategies

**DOI:** 10.3389/fcell.2022.830208

**Published:** 2022-03-08

**Authors:** Kevin Park, Mysore S. Veena, Daniel Sanghoon Shin

**Affiliations:** ^1^ Department of Medicine, Division of Hematology/Oncology, Los Angeles, CA, United States; ^2^ VA Greater Los Angeles Healthcare System, University of California, Los Angeles (UCLA), Los Angeles, CA, United States; ^3^ Molecular Biology Institute, Los Angeles, CA, United States; ^4^ Jonsson Comprehensive Cancer Center, Los Angeles, CA, United States

**Keywords:** tumor microenvironment, cancer, immune escape, immune checkpoint inhibitors, immunotherapy, clinical trials

## Abstract

The tumor microenvironment (TME) is a complex, dynamic battlefield for both immune cells and tumor cells. The advent of the immune checkpoint inhibitors (ICI) since 2011, such as the anti-cytotoxic T-lymphocyte associated protein (CTLA)-4 and anti-programmed cell death receptor (PD)-(L)1 antibodies, provided powerful weapons in the arsenal of cancer treatments, demonstrating unprecedented durable responses for patients with many types of advanced cancers. However, the response rate is generally low across tumor types and a substantial number of patients develop acquired resistance. These primary or acquired resistance are attributed to various immunosuppressive elements (soluble and cellular factors) and alternative immune checkpoints in the TME. Therefore, a better understanding of the TME is absolutely essential to develop therapeutic strategies to overcome resistance. Numerous clinical studies are underway using ICIs and additional agents that are tailored to the characteristics of the tumor or the TME. Some of the combination treatments are already approved by the Food and Drug Administration (FDA), such as platinum-doublet chemotherapy, tyrosine kinase inhibitor (TKI) -targeting vascular endothelial growth factor (VEGF) combined with anti-PD-(L)1 antibodies or immuno-immuno combinations (anti-CTLA-4 and anti-PD-1). In this review, we will discuss the key immunosuppressive cells, metabolites, cytokines or chemokines, and hypoxic conditions in the TME that contribute to tumor immune escape and the prospect of relevant clinical trials by targeting these elements in combination with ICIs.

## Introduction

The tumor microenvironment (TME) is composed of tumor cells, various associated cells of the host, and surrounding extracellular matrix components consisting of various cytokines, chemokines, proteases, many enzymes, microvesicles, and other secreted molecules. The host cell population of the TME mainly consists of fibroblasts, endothelial cells, granulocytes, lymphocytes, and macrophages (Mantovani et al., 2008). Constant spatio-temporal changes in the TME composition are highly complex in nature as the tumor advances in time. In addition to adapting to the changing TME, cancer cells escape destruction by the host immune system by manipulating their own immunogenicity, producing immunosuppressive mediators, and attaining immunomodulatory phenotypes.

To circumvent immunosuppression, immunotherapy rose to stardom since the approval of the first immune checkpoint inhibitor (ICI), ipilimumab, in 2011. Ipilimumab is a monoclonal antibody (mAb) targeting cytotoxic T-lymphocyte associated protein (CTLA-4), demonstrating increased lymphocyte counts and CD4^+^/CD8^+^ T cell percentages in melanoma patients correlated to improved survival (Martens et al., 2016). Since its initial success in melanoma patients, it has received Food and Drug Administration (FDA) approvals in combination with nivolumab (anti-programmed cell death receptor (PD-1) antibody) for the treatment of poor-risk advanced renal cell carcinoma (RCC), microsatellite instability-high or mismatch repair deficient metastatic colorectal cancer (CRC), hepatocellular carcinoma (HCC) previously treated with sorafenib and advanced non-small cell lung cancer (NSCLC) or malignant pleural mesothelioma. Thus, the era of immunotherapy began, indicated by the 18% decrease in the overall mortality rate for metastatic melanoma from 2013 to 2016 - a trend attributable to the effects of ICI therapies (Cable et al., 2021).

However, ICI monotherapy suffers from low response rates of about 13% and moderately high rates of immune-related adverse events, with incidences of 72 and 66% in anti-CTLA-4 and anti-PD-(L)1 therapies, respectively (Sun and Lu, 2020). In 2019, a retrospective, cross-sectional study found that about 39% the U.S. population of cancer patients were eligible for immunotherapy treatment (Haslam et al., 2020). This unfavorable outlook is attributable to the multidimensional TME which continuously devises additional mechanisms of resistance, limiting both initial and prolonged responses to immunotherapies (Bagchi et al., 2021).

To enhance both the response rates and number of candidates for ICI therapies, a comprehensive understanding of the TME is imperative to tackle the resistance to ICI therapies exhibited by the majority of patients. Many clinical trials today are investigating the combination of different immunotherapies or immunotherapy and chemotherapy together in a multifaceted approach, targeting more than one ICIs within the TME to maximize response rates and circumvent resistance (Bagchi et al., 2021). Beyond combination regimens involving ICIs, multi-agent treatments involving the use of therapeutic agents to disable the immunosuppressive cells contributing to ICI resistance is expected to greatly augment immunotherapy. In this review we will discuss the key players contributing to the immunosuppressive TME, the possibility and potential of combination regimens involving multiple cell types, and current clinical trials being conducted to target these aspects within the TME.

## CD8^+^ Cytotoxic T Cells

**FIGURE 1:**
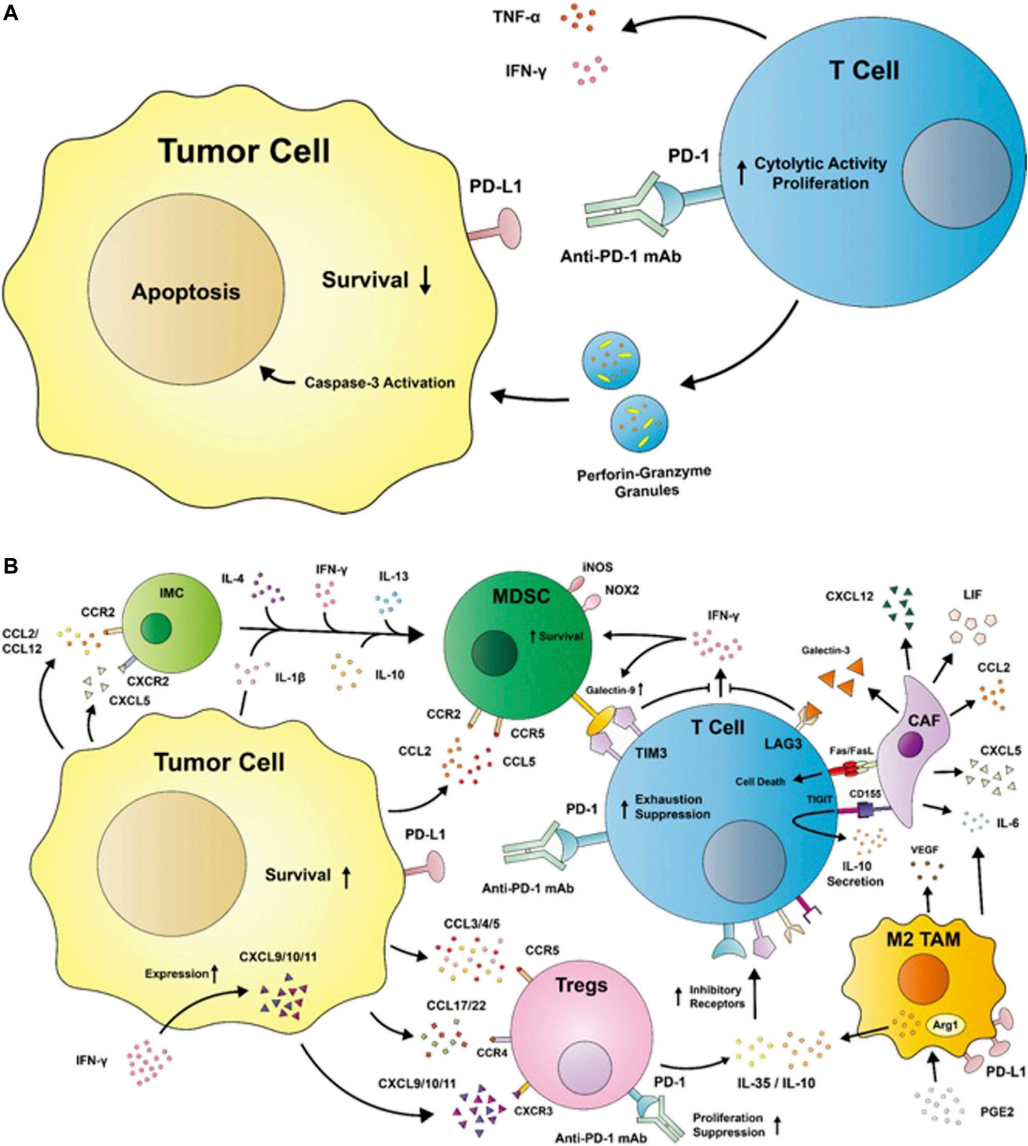
**(A)** A diagram depicting the restoration of effector T cell antitumor activities by immune checkpoint inhibitors such as the anti-programmed cell death protein 1 (PD-1) monoclonal antibody. T cells secrete cytokines such as tumor necrosis factor (TNF) -α and interferon (IFN) -γ to generate an inflammatory environment while releasing granules with perforin and granzyme B to induce tumor apoptosis. **(B)** Immunosuppression in the TME. Tumor cells release chemokines such as C-C motif ligands (CCLs) and C-X-C motif ligands (CXCLs), which interact with C-C motif receptors (CCRs) and C-X-C motif receptors (CXCRs), respectively, to recruit immunosuppressive cells such as regulatory T cells (Tregs), myeloid-derived suppressor cells (MDSCs), M2-like tumor-associated macrophages (TAMs), and cancer-associated fibroblasts (CAFs) into the tumor microenvironment. Tregs secrete interleukin (IL)-35 and IL-10 to induce the upregulation of inhibitory receptors such as PD-1, T cell immunoglobulin and mucin-domain containing 3 (TIM3), T cell immunoglobulin and immunoreceptor tyrosine-based inhibitory motif domain (TIGIT), and lymphocyte activation gene 3 (LAG3). The binding of anti-PD-1 mAb to Tregs has the potential to increase its suppressive functions. The engagement of inhibitory receptors impairs T cell antitumor activities by suppressing IFN-γ and inducing T cell exhaustion by promoting its secretion of anti-inflammatory cytokines. M2-like TAMs are induced by the prostaglandin E2 (PGE2)—abundant TME to upregulate the expression of arginase 1 (Arg1) and IL-10, and secrete factors like IL-6 and vascular epithelial growth factor (VEGF) to promote tumor immune escape and migration. Tumors recruit both immature myeloid cells (IMCs) and MDSCs into the TME, where the expansion of IMCs into MDSCs are induced by factors such as IL-1β, IL-4, IL-10, IL-13, and IFN-γ. MDSCs exert immunosuppression via the expression of the TIM3 ligand galectin-9, inducible nitric oxide synthase (iNOS), and nicotinamide adenine dinucleotide phosphate oxidase (NOX) 2. iNOS and NOX2 produce nitric oxide and reactive oxygen species, which are detrimental to proper immune functions. CAFs secrete a variety of factors such as CXCL5, CXCL12, CCL2, and leukemia inhibitory factor (LIF), which serve immunosuppressive functions. CAFs are additionally capable of inducing T cell death with Fas ligand (FasL). CCR: C-C motif receptor; CD: cluster of differentiation; PD-L1: programmed cell death protein ligand 1.

Cytotoxic CD8^+^ T cells are undoubtedly the major players in immunotherapy today, carrying out cytolytic activities against tumor cells. Effector CD8^+^ T cell activation is dependent on the recognition of an antigen-major histocompatibility complex (MHC) on a tumor cell; upon successful recognition, CD8^+^ T cells release granules that contain perforin, granzyme, and the Fas ligand into the immunological synapse to carry out effector functions (Figure 1A) (Iwahori, 2020). CD8^+^ T cells exist in different cytotoxic T cell (Tc) subsets: the Tc1 subset is responsible for the aforementioned production of granzyme B, perforin, and cytokines such as IFN-γ and tumor necrosis factor (TNF)-α. Tc2 cells do not produce as much IFN-γ as Tc1 cells but maintain a comparable level of cytotoxic activities via granzyme B, while Tc22 similarly express granzyme B to provide antitumor activities and comprise up to 35% of expanded effector T cells from tumors (St Paul et al., 2020; St Paul and Ohashi, 2020). Contrastingly, the Tc9 and Tc17 subsets demonstrate poor cytolytic functions due to their low levels of granzyme B (St Paul and Ohashi, 2020). Tc polarization can be affected by a variety of factors: tumor-associated macrophages (TAMs) from HCC patients were shown to induce Tc17 polarization *in vitro*, intestinal dendritic cells (DCs) were found to induce Tc9 polarization upon antigen cross-presentation, and Langerhans cells were shown to induce Tc22 polarization (Fujita et al., 2009; Kuang et al., 2010; Chang et al., 2013). While the role of the TME in driving Tc cell polarization has yet to be described, the composition of Tc subsets within a tumor can serve as a significant factor determining the response rates to ICI therapies.

CD8^+^ T cells additionally express immune checkpoint molecules to limit their functions, a mechanism harnessed by tumor cells to induce T cell exhaustion and promote cancer progression (Raskov et al., 2021). In an attempt to revitalize the T cell’s effector functions, interleukin (IL)-2 therapy was approved by the FDA for the treatment of renal cell carcinoma and metastatic melanoma in 1992 and 1998, respectively (Wrangle et al., 2018). Interleukin (IL)-2 signaling is required to sustain the effector T cell (Teff)’s cytolytic activity and induce their proliferation (Pipkin et al., 2010); however, a recent study by Liu et al. revealed IL-2’s newfound role in driving T cell exhaustion instead, uncovering its inhibitory potential (Liu et al., 2021).

### Co-Inhibitory Molecules and Related Therapies

Immunotherapy today instead relies on inhibitors to block signals leading to T cell dysfunction, effectively removing the restraints on the effector cells (Figure 1A). While ICI therapies have displayed promising results, they struggle heavily from limited accessibility and performance. In a retrospective cross-sectional study by Haslam et al. from 2011 to 2019, 36.1–38.5% of U.S. patients with cancer were estimated to be eligible for ICIs while the response rates to the therapies were projected to be 10.9–11.4% (Haslam et al., 2020).

To improve the efficacy of ICIs, combination therapies using both anti-PD-1 and anti-CTLA-4 checkpoint inhibitors are approved for few types of cancers and under investigation for several others. As reviewed by Toor et al. (2020) and Jiang et al. (2019), CTLA-4 and PD-1 are the most targeted inhibitory pathways involved in the immune escape by tumors. In patients with previously untreated unresectable stage III or IV melanomas, nivolumab plus ipilimumab demonstrated greater objective response rate (ORR), median overall survival (OS) and progression-free survival (PFS) than ipilimumab alone (Wolchok et al., 2021). Similarly, in patients with ovarian cancer, nivolumab plus ipilimumab exhibited both increased ORR and PFS than nivolumab alone within 6 months of enrollment (Zamarin et al., 2020). Given these promising results, ipilimumab in combination with nivolumab has gained FDA approval for the treatment of several cancer types, such as melanoma, advanced RCC, CRC, HCC, metastatic NSCLC, and malignant pleural mesothelioma (Ipilimumab FDA, 2021). Combination therapy, however, is not without its limitations; for example, the relatively brief PFS reported by Zamarin et al. indicates its short-lived benefits (Zamarin et al., 2020). Likewise, while the Checkmate 032 trial (NCT01928394) in recurrent small-cell lung cancer (SCLC) patients revealed a greater ORR for the nivolumab plus ipilimumab group than nivolumab alone, it failed to replicate the same trends for both OS and PFS (Ready et al., 2020). The increased toxicity due to the combination regimen is also a major concern. Patients receiving combination treatments have higher, more severe incidences of treatment-related adverse events (TRAEs) than single-agent treatments, although the respective TRAEs are generally manageable (Warner and Postow, 2018; Cheng et al., 2020; Wolchok et al., 2021). Ready et al. proposed the discontinuation of the combination treatment due to increased toxicity as a possible explanation for the similar OS and PFS between the two groups, but their claims have not been verified (Ready et al., 2020).

Currently, clinical trials are evaluating the efficacy of ipilimumab and nivolumab combination in various cancer types to expand its use for the treatment of breast cancer, esophageal cancer, head and neck cancer, Hodgkin’s lymphoma, and SCLC (Kooshkaki et al., 2020). Research into different approaches to minimize side effects and increase response rates are undergoing as well. For instance, the first-in-human phase I clinical trial of CRISPR–Cas9 (clustered regularly interspaced short palindromic repeats associated with Cas9 endonuclease) - engineered T cells prevent the expression of co-inhibitory molecules by deleting the *TRAC*, *TRBC* and *PDCD1* loci, demonstrating the potential of CRISPR gene-editing for immunotherapy (NCT04417764 (Wang,MD, 2020) and NCT03525782 (Chen, 2018)) (Stadtmauer et al., 2020; Sun and Lu, 2020). Further studies are warranted to increase the duration of clinical benefits from the combination therapies by incorporating additional agents, and to determine whether the discontinuation of treatment due to the increased toxicity of combination regimens has a significant effect on clinical outcomes.

Combination therapy is appealing in its ability to target more than one immune checkpoint molecule, leaving tumor cells with less options for immune escape. As such, additional inhibitory receptors contributing to T cell dysfunction in the TME have been identified. T cell immunoglobulin and mucin-domain containing 3 (TIM3) promotes CD8^+^ T cell apoptosis or exhaustion upon binding to its ligands, galectin-9 (gal-9), phosphatidylserine, high-mobility group protein B1, and carcinoembryonic antigen cell adhesion molecule 1 (Huang et al., 2015; Kang et al., 2015; Acharya et al., 2020). Gal-9 is produced by cells such as T cells, B cells, macrophages, gastrointestinal epithelial cells, endothelial cells, and fibroblasts, and is upregulated in response to IFN-γ (Figure 1B). Binding of gal-9 releases Bat3 from the intracellular tail of TIM3 to ultimately result in T cell inhibition. Carcinoembryonic antigen cell adhesion molecule 1 also similarly releases Bat3 from TIM3 upon binding to TIM3 to inhibit T cell receptor (TCR) signaling, and is expressed on activated T cells, DCs, monocytes, macrophages, and tumor cells (Acharya et al., 2020). TIM3 expression is driven by IL-27, which engages the IL-27 nuclear factor, interleukin 3-regulated axis to cooperate with T-bet and induce IL-10 expression by T cells (Zhu et al., 2015).

Targeting TIM3 has promising potential to overcome resistance acquired after initial immunotherapy. The most abundant tumor infiltrating lymphocyte (TIL) population in multiple solid tumors were found to be CD8^+^ TILs expressing both TIM3 and PD-1, and targeting both co-inhibitory molecules was shown to rescue exhausted CD8^+^ T cells (Tian and Li, 2021). T cells in murine models of lung adenocarcinoma upregulated TIM3 expression, displaying a positive correlation with the duration of PD-1 blockade administered to the mice. TIM3 blockade following anti-PD-1 resistance increased survival, suggesting the upregulation of TIM3 surface expression as a means of ICI resistance (Koyama et al., 2016). Hence, by identifying and targeting additional co-inhibitory molecules in the TME, more durable ORRs can be expected. The development of TIM3 mAbs are currently under investigation for use with anti-PD-1/PD-L1 therapies. The combination therapy of TIM3 and PD-1/PD-L1 monoclonal antibodies, cobolimab and dostarlimab, respectively, have shown promising results in phase I trials of NSCLC patients resistant to anti-PD-1/PD-L1 therapy alone, displaying increased clinical activity in addition to manageable toxicity (Wolf et al., 2020). Phase II and III trials of the combination therapy are currently ongoing to validate its efficacy and safety (NCT04655976 (GlaxoSmithKline, 2021)) Table 1.

**TABLE 1:** List of current cancer immunotherapy clinical trials.

Treatment	Targets	Disease	Phase	Trial identifier
T Cell-based Therapies				
nivolumab or nivolumab/ipilimumab or nivolumab/ipilimumab/cobimetinib	PD-1, CTLA-4, MEK pathway	Advanced/Metastatic Solid Tumors	I/II	NCT01928394
PD-1 knockout engineered T cell	PD-1	Advanced HCC	I	NCT04417764
Anti-MUC1 CAR T cells and/or PD-1 knockout engineered T cells in comparison to nivolumab	PD-1	Advanced NSCLC	I/II	NCT03525782
cobolimab, dostarlimab, Docetaxel	TIM3, PD-1	Advanced NSCLC	II/III	NCT04655976
etigilimab and nivolumab	TIGIT, PD-1	Advanced/Metastatic Solid Tumors	I/II	NCT04761198
etigilimab and nivolumab	TIGIT, PD-1	Platinum-Resistant Recurrent CCO, PP, or FT Cancer	II	NCT05026606
pembrolizumab and vibostolimab	PD-1, TIGIT	Metastatic NSCLC	III	NCT04738487
BMS-986207, nivolumab, and ipilumumab	TIGIT, PD-1, CTLA-4	Solid Tumors	I/II	NCT02913313
MGD013 (tebotelimab)	PD-1, LAG3	Melanoma	I	NCT04653038
FS118	PD-1, LAG3	SCCHN	I/II	NCT03440437
Treg-based Therapies				
RO7296682 and atezolizumab	CD25, PD-L1	Advanced Solid Tumors	I	NCT04642365
ATOR-1015	CTLA-4, OX40	Solid Tumors	I	NCT03782467
ADCT-301	CD25	AML, MDS, or MDS/MPN	II	NCT04639024
ADCT-301 and pembrolizumab	CD25, PD-1	Advanced Solid Tumors	I	NCT03621982
MDSC-based Therapies				
SX-682 and pembrolizumab	CXCR2, PD-1	Metastatic Melanoma	I	NCT03161431
durvalumab with AZD9150 or AZD5069	PD-L1, STAT3, CXCR2	Advanced Solid Tumors, Relapsed Metastatic SCCHN	II	NCT02499328
pexidartinib (PLX3397)	CSF-1R	Tenosynovial Giant Cell Tumor	III	NCT04488822
Entinostat and pembrolizumab	HDAC, PD-1	Relapsed and Refractory Lymphoma	II	NCT03179930
Entinostat and pembrolizumab	HDAC, PD-1	Stage III/IV Melanoma	II	NCT03765229
TAM-based Therapies				
TTI-621 and rituximab or nivolumab	SIRPα, CD20, PD-1	Hematologic Malignancies and Solid Tumors	I	NCT02663518
IPI-549 and nivolumab	PI3Kγ, PD-1	Advanced Urothelial Carcinoma	II	NCT03980041
IPI-549 and nivolumab	PI3Kγ, PD-1	Advanced Solid Tumors	I	NCT02637531
IPI-549 and atezolizumab/Paclitaxel/bevacizumab	PI3Kγ, PD-L1, VEGF	Triple-Negative Breast Cancer or RCC		NCT03961698
CAF-based Therapies				
Paricalcitol, Gemcitabine, and Nab-paclitaxel	VDR	Metastatic Pancreatic Cancer	I/II	NCT03520790
Paricalcitol, Gemcitabine, and Nab-paclitaxel	VDR	Advanced Pancreatic Cancer	II	NCT04617067
Olaptesed pegol (NOX-A12), pembrolizumab and Nanoliposomal Irinotecan or Gemcitabine/Nab-Paclitaxel	CXCL12, PD-1	Metastatic Pancreatic Cancer	II	NCT04901741
Hypoxia-based Therapies				
Evofosfamide and ipilimumab	CTLA-4	Pancreatic Cancer, Melanoma, SCCHN, Prostate Cancer	I	NCT03098160
Hyperbaric oxygen therapy and camrelizumab	PD-1	Advanced/Metastatic HCC	I	NCT05031949
Exosome-based Therapies				
Mesenchymal Stromal Cells-derived Exosomes with KRAS G12D siRNA	KRAS	Metastatic Pancreatic Cancer	I	NCT03608631

HCC: hepatocellular carcinoma, NSCLC: Non-small cell lung cancer, MUC1: Mucin 1, CAR: chimeric antigen receptor, CCO: clear cell ovarian, PP: primary peritoneal, FT: fallopian tube, AML: acute myeloid leukemia, MDS: myelodysplastic syndrome, MDS/MPN: myeloproliferative neoplasm, SCCHN: Squamous cell carcinoma of head and neck HDAC: Histone deacetylase RCC: renal cell carcinoma, VDR: Vitamin D Receptor, MEK: Mitogen-activated Extracellular kinase, SIRP α: Signal Regulatory Protein α, PI3Kγ: Phosphoinositide 3-kinase γ, VEGF: vascular endothelial growth factor, CXCL: Chemokine (C-X-C) motif ligand, CXCR: Chemokine (C-X-C) motif receptor, CD: cluster of differentiation, CSF-1R: Colony Stimulating Factor 1 Receptor, STAT3: Signal Transducer And Activator Of Transcription 3.

T cell immunoglobulin and immunoreceptor tyrosine-based inhibitory motif domain (TIGIT) is expressed on CD4^+^ and CD8^+^ T cells, natural killer (NK) cells, and regulatory T cells (Tregs), where its expression is upregulated following activation (Harjunpää and Guillerey, 2020). Binding of TIGIT to CD155 (poliovirus receptor PVR) expressed on tumor cells results in the decreased secretion of pro-inflammatory cytokines such as IFN-γ, IL-17a, and TNF-α, and an increased secretion of the anti-inflammatory cytokine IL-10 (Figure 1B) (Zhang C. et al., 2020). TIGIT inhibits T cell function by decreasing T cell activation, IL-2 production, and TCR-mediated proliferation (Lozano et al., 2012). TIGIT’s receptor, CD155, is highly expressed on DCs, fibroblasts, endothelial cells and tumor cells within the TME, creating a highly immunosuppressive environment (Figure 1B). The engagement of the TIGIT/CD155 axis by T cells and DCs, respectively, inhibits T cell functions by inducing a tolerogenic DC phenotype. TIGIT’s competing receptors to CD155 include CD226 (DNAM-1) and CD96, both of which have lower affinity to CD155 than TIGIT (Yu et al., 2009). CD226 especially has potential positive effects on T cells upon engagement to CD155 by promoting T-bet-mediated IFN-γ production. However, TIGIT’s higher affinity for CD155 impedes the anti-tumoral CD226/CD155 axis, inhibiting anti-tumoral activities through competition (Lozano et al., 2012).

Anti-TIGIT treatments demonstrated efficacy in preclinical trials of multiple myeloma and head and neck squamous cell carcinoma (HNSCC) by decreasing tumor progression in a CD8^+^ T cell-dependent manner (Guillerey et al., 2018; Wu et al., 2019). Targeting the TIGIT/CD155 axis is appealing as TIGIT and CD155 were each found to be highly expressed by Tregs and MDSCs, respectively, where CD155s expression by stromal or epithelial cells in particular was associated with worse survival in HNSCC patients (Wu et al., 2019). Etiglimab is an anti-TIGIT mAb currently undergoing clinical trials in combination therapy with nivolumab for the treatment of locally advanced or metastatic tumors, in addition to platinum-resistant carcinoma (NCT04761198 (Mereo BioPharma, 2021) and NCT05026606 (M.D. Anderson Cancer Center, 2021a)). Etiglimab demonstrated promising results in preclinical trials by preventing human melanoma growth in mice reconstituted with human hematopoietic stem cells. Other monoclonal antibodies to TIGIT, such as vibostolimab, domvanalimab, BMS-986207, and ASP8374 are in phase I and II clinical trials to confirm their efficacies and safety profiles in monotherapy or combination therapies with pembrolizumab, nivolumab, ipilimumab, pemetrexed, or carboplatin (NCT04738487 (Merck Sharp and Dohme Corp., 2021) and NCT02913313 (Bristol-Myers Squibb, 2021)) (Harjunpää and Guillerey, 2020). Recent updates on the trials testing anti-TIGIT with anti-PD-(L)1 combination treatments appear to be promising; it is highly anticipated that these combination treatments may able to provide improvement in response rates (Rodriguez-Abreu et al., 2020; Chau et al., 2021) Table 1.

Exhausted CD8^+^ T cells express the lymphocyte activation gene 3 (LAG3/CD223) in response to prolonged activation (Huard et al., 1994). In melanoma, LAG3 can bind to MHC-IIs upregulated on tumor cells, upregulating MAPK/Erk and phosphatidylinositol-3-kinase (PI3K)/Akt pathways to confer melanoma cells resistance to Fas-mediated and drug-induced apoptosis (Hemon et al., 2011). LAG3-MHC-II binding additionally recruits tumor-specific CD4^+^ T cells, decreasing the CD8^+^ T cell response (Donia et al., 2015). Galectin-3 (gal-3) is another LAG3 ligand expressed by epithelial, myeloid, and stromal cells, including cancer-associated fibroblasts (CAFs) (Dumic et al., 2006; Dong et al., 2018). Binding of gal-3 to LAG3 on CD8^+^ T cells leads to the decreased expression of pro-inflammatory cytokines such as IFN-γ, TNF-α, and IL-6 (Figure 1B) (Kouo et al., 2015). Other LAG3 ligands include LSECtin expressed on DCs and fibrinogen-like protein 1, which similarly leads to the inhibition of IFN-γ secretion by Teffs, suppression of IL-2 induction, and TNF-α and IFN-γ secretion (Xu et al., 2014; Wang et al., 2019).

Monoclonal antibodies to LAG3, such as relatlimab (BMS-986016), LAG525, BI754111, MK-4280, Sym022, TSR-033, REGN3767, and INCAGN2385-101 are currently undergoing phase I and II clinical trials as monotherapy or combination therapy with anti-PD-1/PD-L1 mAbs to treat multiple myeloma, SCLC, NSCLC, gastric/esophageal adenocarcinoma, and CRC. Antagonistic bispecific antibodies to LAG3, such as MGD013, FS118, and xmab22841, respectively targeting PD-1, PD-L1, and CTLA-4 in addition to LAG3 are also in clinical trials (NCT04653038 (Zai Lab (Shanghai) Co., Ltd., 2020) and NCT03440437 (F-star Therapeutics Limited, 2021)) Table 1. Murine models of chronic lymphocytic leukemia (CLL) treated with both anti-PD-1 and anti-LAG3 displayed a significantly lower number of CLL cells in the spleen, along with a decrease in Tregs and an increase in Teffs (Wierz et al., 2018). Relatlimab in combination with nivolumab has shown efficacy in melanoma patients with LAG3 expression in at least 1% of tumor-associated immune cells, demonstrating an ORR of 18% (n = 33). In contrast, melanoma patients with LAG3 expression in less than 1% of their immune cells had an average ORR of 5% (n = 22), indicating that LAG3 inhibition therapy may be a specific, targeted therapy for patients with high expression of LAG3 in their TME (Ascierto et al., 2017). Although the combination therapy of anti-LAG3 and anti-PD-1/PD-L1 mAbs have resulted in durable responses in 9.9% of patients (n = 121), the exact mechanism contributing to the synergy is unknown (Maruhashi et al., 2020).

Targeting LAG3 faces unique challenges due to the different role of soluble LAG3 (sLAG3). sLAG3 carries out antitumoral activities by allowing DCs to mature and attack tumor cells. sLAG3 exhibits a positive correlation with CD8^+^ T cells, secretion of IL-12 and IFN-γ, and survival in murine models of gastric cancer (GC) (He et al., 2016; Li et al., 2018, 3). These characteristics of sLAG3 opens avenues for its potential use as a therapeutic agent for certain cancer types, but also indicates the possibility of LAG3 inhibitors interacting with sLAG3. Therefore, while LAG3 inhibitors are promising treatment options, their molecular mechanisms of action in combination regimens with PD-1, their effects on sLAG3, and mechanisms of resistance to anti-LAG3 therapies warrant further investigation (Barrueto et al., 2020).

## Regulatory T Cells

Characterized as CD4^+^CD25^+^FoxP3^+^ T cells, Tregs are responsible for carrying out immunosuppressive activities in the TME to drive tumor progression. IL-10 secretion by Tregs inhibits the production of pro-inflammatory cytokines such as TNF-α, IL-1β, IL-6, and IL-12 in macrophages in a signal transducer and activator of transcription (STAT) 3-dependent pathway (Lang et al., 2002), and suppresses downstream signaling of CD28 in T cells via the Janus kinase (JAK) 1 and tyrosine kinase 2 pathways (Taylor et al., 2006). Tregs additionally secrete IL-35, a member of the IL-12 family that inhibits CD4^+^ and CD8^+^ Teff proliferation via the IL-35 receptor-mediated activation of STAT4 and STAT1. IL-10 and IL-35 additionally increases the expression of inhibitory molecules on CD8^+^ T cells, making them susceptible to exhaustion and suppression (Figure 1B) (Sawant et al., 2019). IL-35 has the ability to induce a regulatory population of T cells that do not express FoxP3, which exhibits potent immunosuppressive capacities *in vivo* (Collison et al., 2010). Upon induction, Tregs rely on IL-2 for their maturation and function, using CD25 as the receptor for the cytokine. Because Tregs are unable to produce IL-2, Tregs consume large quantities of IL-2 from the TME and impair T cell functions by depriving the cytokine from the TME (Li C. et al., 2020).

Tregs rely on the C-C motif receptor (CCR) 4/C-C motif ligand (CCL) 22 or CCR4/CCL17 pathway for chemotaxis (Figure 1B). In a study by Curiel et al. (2004), tumor Tregs with high expressions of CCR4 interacted with CCL22 or CCL17 secreted by tumor cells or TAMs to facilitate their recruitment into the TME (Iellem et al., 2001; Curiel et al., 2004; Dadey et al., 2020). These CCR4^+^ tumor Tregs have been characterized as the most suppressive subset, allowing them to abundantly infiltrate tumor sites in multiple cancers such as gastric and esophageal cancers (Sugiyama et al., 2013). Tumor cells and MDSCs in the TME are able to secrete CCL3, CCL4, and CCL5, interacting with CCR5 on Tregs and attracting them into the TME (Tan et al., 2009; Schlecker et al., 2012). The pro-inflammatory conditions in the TME created by IFN-γ further promotes Treg recruitment by inducing the expression of C-X-C motif ligand (CXCL) 9, CXCL10, and CXCL11, all three of which are ligands for C-X-C motif receptor (CXCR) 3 (Müller et al., 2007; Redjimi et al., 2012; Tokunaga et al., 2018). CXCR3 is expressed on Tregs via T-BET and similarly induced by the pro-inflammatory conditions within the TME (Koch et al., 2009).

Tregs are capable of synergizing with other immunosuppressive immune cells within the TME, such as the myeloid-derived suppressor cells (MDSCs). CD80/CTLA-4 interactions between MDSCs and Tregs, respectively, can augment the suppressive functions of Tregs (Yang et al., 2006), while IL-35 secreted by Tregs can induce MDSC accumulation and increase immunosuppressive effects in the TME (Wang Z. et al., 2021). Tregs also express the nucleases CD39 and CD73 on their surfaces, catalyzing the conversion of ATP or ADP into AMP and AMP into adenosine, respectively (Li C. et al., 2020). Adenosine binds to the A2A adenosine receptor (A2AR) expressed on the surface of immune cells that evoke different responses depending on the target. On CD4^+^ and CD8^+^ T cells, the engagement of the A2AR-mediated pathway leads to the inhibition of T cell function by restricting their proliferation, cytotoxicity, and cytokine secretion (Ohta et al., 2012). In contrast, A2AR stimulation on Tregs not only leads to their proliferation, but also inhibits the IL-2 production by Teffs (Zarek et al., 2008; Ohta et al., 2012).

OX40 is a co-stimulatory molecule part of the tumor necrosis factor receptor superfamily expressed on the surfaces of activated T cells, neutrophils, and NK cells. Its receptor, OX40L (CD252), is expressed on antigen-presenting cells such as DCs, activated B cells, and macrophages. In Tregs, OX40 expression is constitutive and its ligation dampens their suppressive abilities by limiting their proliferation and generation of IL-10 producing type 1 Tregs (Fu Y. et al., 2020; Kuang et al., 2020). However, in a study of HCC patients with and without cirrhosis, it has been implied that OX40 signaling may contribute towards the survival and proliferation of Tregs. The cirrhotic microenvironment can induce the development of highly suppressive OX40^+^ Tregs, where OX40L^+^ TAMs provide signals to expand Tregs and promote the development of HCC from cirrhosis (Piconese et al., 2014). In a later study in HCC patients, HCC tumors were shown to express high levels of OX40, where IL-2 could upregulate OX40 expression and interact with the molecule to drive Treg proliferation. In HCC tumors with high OX40 expression, despite the greater activation of Teffs, they do not correlate to increased antitumor activities, suggesting the involvement of functionally defective CD8^+^ T cells or immune checkpoint molecules (Xie et al., 2018). Additional work to characterize the aspects of the HCC TME contributing to the conflicting role of OX40 would be of great benefit to fully understand its effects on Tregs.

Like T cells, Tregs express immune checkpoint molecules on their surfaces. Tregs can target DCs with co-inhibitory receptors such as CTLA-4 and LAG3 (Li C. et al., 2020). Tregs constitutively express CTLA-4 on their surfaces (Read et al., 2000), impairing the upregulation of CD80 and CD86 on DCs and limiting the activation of naive T cells by inhibiting CD28 signaling (Wing et al., 2008). Binding of CTLA-4 to CD80/CD86 on the surfaces of DCs also upregulates indoleamine 2,3-dioxygenase (IDO) to produce kynurenine, a metabolite of tryptophan that suppresses Teff function and promotes Treg synthesis (Mellor and Munn, 2004; Li C. et al., 2020). Upon Treg activation and in the presence of Teffs, Tregs upregulate the expression of LAG3, which is essential for their immunosuppressive functions (Huang et al., 2004). Increased frequencies of LAG3^+^ Tregs were found in the PBMCs of cancer patients, which expand in the TME to secrete IL-10 and transforming growth factor beta 1 (TGF-β1) (Camisaschi et al., 2010). However, in the context of Type 1 diabetes, LAG3^+^ Tregs exhibited limited proliferation and function, contributing to autoimmunity by failing to induce immunosuppression (Zhang et al., 2017). Further studies into LAG3’s role in Treg function are required to elucidate the mechanisms behind its variable activity in the tumor and autoimmune settings.

TIM3 and PD-1 are co-inhibitory molecules often co-expressed on the surfaces of Tregs. TIM3^+^ Tregs exhibited higher suppressive capacities relative to TIM3^-^ Tregs towards T helper (Th) 1 and Th17 cells, with TIM3 expression being correlated to worse prognosis in lung cancer (Gao et al., 2012; Gautron et al., 2014). Similarly, TIGIT activation exerts potent inhibitory effects on Teffs and NK cells, but contrastingly enhances the immunosuppressive activities of Tregs. Ligation of TIGIT on Tregs induces their Fgl2 production and secretion, which suppresses Teff proliferation, promotes Th2 polarization, inhibits Th1 and Th17 activities, and induces IL-10 and IL-4 expression (Joller et al., 2014). In melanoma patients, TIGIT^+^ Tregs exhibited greater immunosuppressive capabilities and stability than TIGIT- Tregs by increasing the expression of CTLA-4, CD39, PD-1, and TIM3 (Fourcade et al., 2018). However, PD-1 expression by Tregs have been shown to limit their proliferation and suppressive capacities (Franceschini et al., 2009), exhibiting an exhausted phenotype marked by increased secretion of IFN-γ (Lowther et al., 2016). This may imply that while anti-PD-1 therapies may restore Teff functions, they may also renew the immunosuppressive abilities of Tregs. In fact, Kamada et al. revealed the presence of highly proliferative, suppressive effector Treg cells in GC patients undergoing hyperprogressive disease after anti-PD-1 treatment, suggesting the therapy’s undesired effects on Tregs (Figure 1B) (Kamada et al., 2019).

### Treg-Based Therapies

In addition to the aforementioned ICI therapies, approaches to disable or deplete immunosuppressive cell populations like Tregs within the TME are pursued to augment the anti-tumoral activities of Teffs Table 1. Like the anti-PD-1 treatment, ICIs may achieve different results on Tregs compared to those on Teffs. For example, anti-CTLA-4 mAbs ipilimumab and tremelimumab were found to increase the density of CD4^+^ and CD8^+^ T cells in tumor tissues but not deplete Tregs (Sharma et al., 2019). By modifying the Fc-region of the anti-CTLA-4 mAb, Ha et al. increased the antibody-dependent cell-mediated cytotoxicity (ADCC) of the inhibitor to deplete activated Tregs highly expressing CTLA-4. However, antigen stimulation results in the expansion of CTLA-4^+^ antigen-specific CD8^+^ T cells, where the high ADCC-CTLA-4 mAb causes the depletion of the Teff population along with Tregs. With the high ADCC-CTLA-4 mAb, the authors recommend the administration of Treg depletion regimen before antigenic stimulation (Ha et al., 2019).

An alternative approach is the anti-CD25 mAb, which binds to CD25 highly expressed on Tregs relative to Teffs. While anti-CD25 mAbs failed to create remarkable responses, Fc-optimized anti-CD25 mAbs were successful in selectively depleting tumor-infiltrating Tregs and increasing the Teff to Treg ratio (Arce Vargas et al., 2017). However, the major concern with anti-CD25 mAb is its ability to interact with IL-2 and block IL-2 signaling crucial to sustain Teffs, resulting in reduced antitumoral activities by decreasing granzyme B expression. Solomon et al. synthesized a modified anti-CD25 mAb, RO7296682 (RG6292), capable of depleting Tregs while preserving IL-2 signaling on Teffs. The non-IL-2 blocking anti-CD25 mAb demonstrated higher activation of CD8^+^ T cells than the Fc-optimized anti-CD25 mAbs, creating synergistic effects with anti-PD-1 therapies in murine models (Solomon et al., 2020). A phase I trial is currently underway with the combination of RO7296682 and atezolizumab to determine its safety and tolerability in patients with advanced solid tumors (NCT04642365 (Hoffmann-La Roche, 2021)).

Strategies to target more than one molecule by the use of bispecific antibodies are rising as an alternative therapeutic option. The pairing of ICIs and co-stimulatory checkpoint molecules is in essence a combination regimen and highly attractive as it can simultaneously drive Teff functions while preventing suppressive cells such as Tregs from depressing the immune response (Galon and Bruni, 2019). ATOR-1015 is a bispecific antibody to CTLA-4 and OX40, which are two receptors highly expressed on Tregs. Agonistic OX40 mAbs were shown to drive Teff proliferation while inhibiting Treg survival and functions in preclinical settings (Voo et al., 2013). ATOR-1015 has shown to induce localized Treg depletion and T cell activation *in vitro* while decreasing tumor growth and improving survival in murine models by enhancing anti-PD-1 treatments. Despite these preclinical findings, the safety profile of ATOR-1015 has yet to be assessed and requires further investigation to evaluate its ability to deplete Tregs (Kvarnhammar et al., 2019). ATOR-1015 is currently undergoing clinical trials to assess its safety and tolerability in patients with solid tumors (NCT03782467 (Alligator Bioscience AB, 2021)). Similarly, there has been the development of the bispecific antibody KY1055, which combines the agonist for inducible T cell co-stimulator and PD-L1-mAb. KY1055 was able to increase the ratio of CD8^+^ Teffs to Tregs and deplete Tregs *in vivo*, but has yet to enter clinical trials (Sainson et al., 2021). Clinical studies comparing the efficacy of agonist-inhibitor bispecific antibodies to single antibodies will help better elucidate the benefits they offer.

Antibody drug conjugates (ADCs) are novel approaches to deliver drugs to targets in a highly specific manner (Dees et al., 2021). A preclinical trial demonstrated the ability of a CD25-targeted, pyrrolobenzodiazepine dimer-based ADC to induce durable antitumor activity by inducing a durable and robust depletion of Tregs. When delivered in combination with anti-PD-1 therapy, there was a greater increase in CD8^+^ TILs, suggesting its synergy with ICIs (Zammarchi et al., 2020). The CD25-targeted, PBD dimer-based ADC, ADCT-301, is currently undergoing phase I clinical trials in patients with lymphoma, leukemia, and various advanced solid tumors (NCT04639024 (Gwynn Long MD, 2021) and NCT03621982 (ADC Therapeutics S.A., 2021)). The importance of precisely targeting Tregs to allow Teffs from exerting antitumoral effects is crucial to augment ICI therapies today, and it is anticipated that combination therapies involving the depletion of Tregs will help achieve greater and prolonged responses.

## Myeloid-Derived Suppressor Cells

MDSCs are another example of immunosuppressive cells within the TME, comprised of heterogeneous, immature myeloid cells (IMCs) generated by emergency myelopoiesis. Emergency myelopoiesis is triggered to meet the high demands of leukocytes generated by pathological conditions, increasing the production of myelomonocytic cells in the bone marrow to supply innate immune cells needed to combat infections (Takizawa et al., 2012). While IMCs normally differentiate into mature neutrophils, macrophages, and DCs upon entering peripheral blood and tissues, tumor-derived soluble factors and pro-inflammatory cytokines in the TME such as IFN-γ, IL-1β, IL-4, and IL-10 instead drive the differentiation of IMCs into MDSCs and later into immunosuppressive macrophages and DCs (Figure 1B) (Bunt et al., 2006; Gallina et al., 2006; Gabrilovich et al., 2012). Tumor cells additionally facilitate the development of MDSCs by secreting CCL2, CCL12, CXCL5 to attract IMCs into the TME while also secreting growth factors that recruit MDSCs in the bone marrow (Huang et al., 2007; Yang et al., 2008; Gabrilovich et al., 2012).

In the TME, MDSCs are activated by tumor-derived TGF-β and drive the proliferation of Tregs (Ghiringhelli et al., 2005). The primary subtypes of MDSCs include polymorphonuclear (PMN) - MDSCs and monocytic (M) - MDSCs. M-MDSCs suppress the immune system via secretion of anti-inflammatory cytokines like IL-10, additionally employing inducible nitric oxide synthase (iNOS) to produce nitric oxide (NO) (Figure 1B). NO has been shown to inhibit the T cell response by promoting T cell apoptosis, decreasing IL-2-mediated signaling, and preventing MHC-II expression. M-MDSCs are recruited to the TME primarily by tumor-derived CCL2 and CCL5, while PMN-MDSCs are recruited by CCL2 and CCL3 (Qian et al., 2011; Reichel et al., 2012; Chun et al., 2015). PMN-MDSCs produce high levels of reactive oxygen species (ROS) with high levels of NADPH oxidase (NOX) 2, resulting in increased recruitment of MDSCs to the TME, disruption of the immune cells’ DNA, and impaired differentiation of MDSCs into DCs. NO and ROS together produce peroxynitrite, which nitrifies both the TCR and T cell-specific chemokines to restrict T cells’ activation and migration (Safarzadeh et al., 2018). Furthermore, both subtypes are capable of producing arginase-1 (arg1), which depletes l-arginine from the TME (Yang et al., 2020) By consuming l-arginine and sequestering l-cysteine from the TME, T cells are deprived of essential amino acids required for their activation, function, and proliferation (Safarzadeh et al., 2018). M-MDSCs are additionally able to provide inhibitory signals by the expression of gal-9, which binds to TIM-3 expressed on CD8^+^ T cells to impair their IFN-γ secretions and confer resistance to anti-PD-1 therapy (Figure 1B) (Limagne et al., 2019).

MDSC expansion in the TME is driven by factors from tumor cells, stromal cells, and immune cells (Safarzadeh et al., 2018). Factors such as Toll-like receptor (TLR) 4, IL-4, IL-1β, TGFβ, and IFN-γ activate pathways such as STAT1, STAT3, STAT6, and NF-kB to sustain MDSC survival and function (Figure 1B) (Gabrilovich and Nagaraj, 2009; Law et al., 2017). One of the main transcription factors regulating MDSC expansion is STAT3, where GM-CSF, macrophage (M)-CSF, and vascular endothelial growth factor (VEGF) activate its downstream signaling. STAT3 is speculated to play a role in driving MDSC proliferation and block the differentiation of IMCs into various mature myeloid cells (Nefedova et al., 2004; Law et al., 2017). Upon STAT3 inhibition, a decrease in MDSC populations and an increase in antitumor activities were observed (Kortylewski et al., 2005). However, Kumar et al. discovered that upon the downregulation of STAT3 activity by hypoxia-induced CD45 protein tyrosine phosphatases, MDSCs are rapidly differentiated into tumor-promoting TAMs (Kumar et al., 2017). While this may raise some concerns about using STAT3 inhibitors to decrease MDSC populations in the TME, a study by Hellsten et al. assessing the performance of galiellalactone, a STAT3 inhibitor, revealed a significant decrease in the generation of M-MDSC but no increase in tumor-promoting TAMs (Hellsten et al., 2019).

### MDSC-Based Therapies

Unlike other cell populations, targeting MDSCs for therapy faces significant challenges due to the cell population being largely heterogeneous with no consensus on the surface markers for MDSC identification (Law et al., 2020). Therefore, various approaches such as disabling the function, recruitment, or expansion of MDSCs by targeting chemokines or signaling pathways have been explored. Because MDSCs exert potent immunosuppressive activities in the TME and contribute towards tumor growth, a combination of both MDSC-targeted therapies and ICIs is becoming increasingly attractive (Table 1). Attempts to use chemotherapeutic agents such as 5-fluorouracil and oxaliplatin, which decrease the MDSC population, in combination with anti-PD-1 therapy in murine models of GC were successful in increasing the antitumor response and Teff tumor infiltration. Kim et al. additionally confirmed that MDSCs contributed to resistance towards anti-PD-1 therapy; during later stages of GC where MDSC accumulation was more significant, anti-PD-1 monotherapy elicited minimal responses compared to its administration during the early stages of GC when less MDSCs were present in the TME (Kim et al., 2021). Therefore, targeting MDSCs prior to or in combination with ICIs is the next step to evoke greater antitumor responses in patients.

Additional therapeutic options to deplete MDSCs include the aforementioned STAT3 inhibitor galiellalactone and the tyrosine kinase inhibitor sunitinib, which in combination decreased MDSC populations from the TME to restore antitumor activities. While sunitinib received FDA approval for the treatment of gastrointestinal stromal tumors, advanced and recurrent RCC, and progressive pancreatic neuroendocrine tumors, galiellalactone has yet to enter clinical trials (Hellsten et al., 2019; Law et al., 2020). An example of a therapeutic strategy aimed towards preventing the recruitment of MDSCs is the CXCR2 inhibitor, which interacts with CXCR2 and CXCR5 (Katoh et al., 2013). CXCR2 inhibition using the small molecule inhibitor SX-682 enhanced the effects of adoptively transferred T cells or NK cells by abrogating MDSC infiltration into the TME (Sun et al., 2019; Greene et al., 2020). SX-682 is currently undergoing clinical evaluation in patients with metastatic melanoma as monotherapy or in combination with pembrolizumab (NCT03161431 (Syntrix Biosystems, Inc., 2021)). Another CXCR2 inhibitor AZD5069 (NCT02499328 (AstraZeneca, 2020, 2)) is currently in phase 1b/2 clinical trial for the treatment of advanced solid tumors and metastatic HNSCC. MDSCs additionally rely on the colony-stimulating factor-1 receptor (CSF-1R)/CSF-1 axis for their recruitment, allowing for the inhibition of CSF-1R as an alternative option. The small molecule inhibitor of CSF-1R, PLX3397 (pexidartinib), led to the increased infiltration of lymphocytes into the tumor with higher IFN-γ secretion (Mok et al., 2014). PLX3397 is currently in phase III for the treatment of tenosynovial giant cell tumor (NCT04488822 (Daiichi Sankyo Co., Ltd., 2021)). However, because anti-CSF-1R therapies suffer from limited therapeutic benefits when administered alone, approaches to use them in combination with ICI therapies or adopted T cell therapies are currently under evaluation and will be discussed later (Ries et al., 2015).

Epigenetic reprogramming is another avenue that can be taken to neutralize MDSCs’ immunosuppressive activities by targeting their effector molecules (Christmas et al., 2018). Entinostat, a class I histone deacetylase inhibitor, was shown to decrease tumor growth and increase survival in murine models of lung cancer and RCC in combination with anti-PD-1/PD-L1 therapy. Orillion et al. discovered that entinostat significantly reduced arg-1, iNOS, and cyclooxygenase-2 (COX-2) to inhibit immunosuppressive activities in the TME, making tumors more susceptible to immune responses by effector cells (Orillion et al., 2017; Law et al., 2020). Clinical trials are currently underway to examine the activity of entinostat in combination with pembrolizumab to treat lymphoma (NCT03179930 (Memorial Sloan Kettering Cancer Center, 2021)) and melanoma (NCT03765229 (UNC Lineberger Comprehensive Cancer Center, 2021)).

## Tumor-Associated Macrophages

As opposed to Tregs or MDSCs, TAMs can support either tumor progression or eradication. Macrophages in the TME exist in a dynamic spectrum of phenotypes across tumor types, dependent on tumor stage and tissue-specific regulation (Li X. et al., 2019). TAMs can be characterized into two major phenotypes: M1 classically activated macrophages and M2 alternatively activated macrophages (Zhang S.-Y. et al., 2020). M1 macrophages can be induced by proinflammatory stimuli, such as IFN-γ, TNF-α, granulocyte-macrophage CSF, and TLR ligands. The transcription factors interferon regulatory factor (IRF) 3 and 5 regulate M1 polarization and the expression of type I interferons. On the other hand, M2 macrophage polarization is driven by immunosuppressive stimuli such as TGF-β, IL-4, IL-10, and IL-13, which activate downstream STAT3 and STAT6 transcription factors (Park-Min et al., 2005; Sinha et al., 2005; Xin et al., 2009; Wang et al., 2014). α-ketoglutarate, the downstream product of glutaminolysis, drives M2-like polarization through fatty acid oxidation (FAO) and Jmjd3-dependent epigenetic reprogramming of M2 genes (Liu et al., 2017).

M1 macrophages promote beneficial anti-tumor effects by driving the Th1 response and via the secretion of TNF-α, IL-1, IL-6, IL-12, Type I IFN, CXCL1-3, CXCL5, and CXCL8-10. M2 macrophages contrastingly perform immunosuppressive functions by supporting the Th2 response and secreting IL-10, TGF-β, CCL17, CCL18, CCL22, and CCL24 (Wang et al., 2014; Wu et al., 2020). M2 macrophages also direct tumor progression via the secretion of adrenomedullin and (VEGF) to promote angiogenesis and expression of PD-L1 to allow immune escape of tumor cells (Figure 1B) (Chen et al., 2011; Jayasingam et al., 2019). TAMs themselves are recruited into the TME by tumor-derived adenosine, the CCL2/CCR2 axis, the CXCL12/CXCR4 axis, and the VEGF receptor pathway; tumor cells, stromal cells, and macrophages are responsible for the production of chemokines necessary to attract TAMs and MDSCs into the TME (Qian et al., 2011; Hughes et al., 2015; Montalbán Del Barrio et al., 2016; Li X. et al., 2019).

Succinate and prostaglandin E2 (PGE2) are abundant molecules in the TME accountable for TAM-dependent tumor progression. Tumor cells have dysfunctions in succinate dehydrogenase, an enzyme involved in tumor suppression. Succinate accumulates in tumor cells due to the dysfunction of succinate dehydrogenase, and high amounts of succinate released by tumor cells into the TME activates succinate receptor 1 (SUCNR1) expressed on macrophages (Selak et al., 2005; Chen et al., 2021). The subsequent activation generates pro-tumor TAMs, driven by the SUCNR1-triggered-PI3K—hypoxia inducible factor (HIF)-1α axis. In return, activated TAMs increase tumor cell migration by secreting IL-6 (Wu et al., 2020). Similarly, high levels of PGE2 in the TME is associated with tumor progression and poor prognosis (Wang and Dubois, 2010). The inflammatory conditions at tumor sites drive the catalysis of PGE2 from arachidonic acid by COX-2 (Mizuno et al., 2019). PGE2 induces M2-like polarization in macrophages via the phosphorylation of cAMP-responsive element binding pathway, increasing CCAAT enhancer binding protein β expression and ultimately upregulating *Arg1*, *IL-10*, and *Mrc1* gene expressions (Figure 1B) (Na et al., 2015). Prima et al. (2017) uncovered PGE2’s role in PD-L1 regulation by showing that inhibitors of COX-2, microsomal PGE2 synthase 1, and overexpression of PGE2-degrading enzyme 15-hydroxyprostaglandin dehydrogenase resulted in decreased PD-L1 expression in TAMs (Prima et al., 2017).

Unlike succinate and PGE2, retinoic acid (RA) is a molecule with ambiguous effects on tumor progression. RA *in vitro* exhibited antitumor properties by decreasing TGF-β1 secretion in tumor cells, impairing their abilities to activate macrophages that secrete VEGF and IL-8 (Liss et al., 2002). All-trans-retinoic acid treatment of prostate cancer cells showed a decreased proliferation of pro-tumoral TAMs, impairing their immunosuppressive capacities by disrupting their cytokine secretion and surface molecule expression (Tsagozis et al., 2014). However, Devalaraja et al. (2020) elucidated the role of tumor cell-derived RA in inhibiting the differentiation of monocytes into immunostimulatory DCs and instead driving the differentiation of pro-tumoral TAMs (Devalaraja et al., 2020). RA downregulates the transcription factor IRF4, and reducing the RA level in the TME induces monocyte differentiation into immunostimulatory antigen-presenting cells and restores T cell activity against tumors. The difference between RA and tumor cell-derived RA leading to contrasting effects need to be better understood.

### TAM-Based Therapies

Along with Tregs and MDSCs, immunosuppressive TAMs are among the main players contributing towards immunotherapy resistance. Treatments to disable the immunosuppressive activities of TAMs in the TME has great potential to be used in combination with ICIs. TAM-targeted therapies today focus on restricting their recruitment, depleting them from the TME, or converting them from M2 to the M1 phenotype Table 1 (Li X. et al., 2019).

Inflammatory cytokines such as IFN-γ and TNF-α induce the production of CSF-1 by tumor cells, which prompts M2 TAM activation or recruitment into the TME (Satriano et al., 1993; Neubert et al., 2018). While anti-CSF-1R treatments demonstrated limited efficacy alone (Ries et al., 2015), anti-CSF1R treatment in combination with anti-PD-1 therapy significantly decreased M2 TAMs while increasing the number of CD4^+^ and CD8^+^ TILs in murine models of melanoma (Neubert et al., 2018). In murine models of mesothelioma, it was demonstrated that mesothelioma tumor cells and CD8^+^ upregulated PD-L1 and PD-1, respectively, in response to anti-CSF-1R therapy alone, emphasizing the importance of incorporating anti-PD-1 therapy into anti-CSF-1R treatment regimens (Magkouta et al., 2021). Anti-CSF-1R therapy moreover was shown to induce the apoptosis of CSF-1R^+^ macrophages, serving as a means to deplete them from the TME (Ries et al., 2014). A clinical trial evaluating the combination of the CSF-1R inhibitor MCS110 and anti-PD-1 mAb PDR001 was recently completed, although a report on its efficacy and tolerability has yet to be published (Novartis Pharmaceuticals, 2021). The inhibition of other TAM-recruiting chemokines such as CCL2 and CXCR4 resulted in decreased tumor growth and progression in preclinical trials; however, their performances in combination with ICIs have not yet been evaluated (Li et al., 2017; Zhou et al., 2018).

Another way of targeting TAMs is to promote their conversion from an immunosuppressive phenotype into an antitumoral, proinflammatory phenotype. Tumor cells express CD47 on their surfaces, which engages the signal regulatory protein alpha (SIRPα) receptor on macrophages to provide a “don’t eat me” signal (Li X. et al., 2019). SIRPα-IgG1 Fc (TTI-621) is a recombinant fusion protein with the SIRPα domain and the Fc domain responsible for providing the prophagocytic signals required for antitumor activities (Ansell et al., 2021). Because TTI-621 demonstrated promising antitumor activities *in vitro* and in murine models of lymphoma (Lin et al., 2017; Petrova et al., 2017), the safety and efficacy of TTI-621 was evaluated in a phase I study as a monotherapy or in combination with rituximab or nivolumab (NCT02663518 (Trillium Therapeutics Inc., 2021)). TTI-621 overall was well-tolerated and elicited objective responses without causing anemia, highlighting its potential as a therapeutic agent using macrophages as its primary effector cells (Ansell et al., 2021). The synergy between SIRPα-IgG1 Fc and anti-PD-1 mAb can be attributed to their combined ability to reverse the M2-like polarization of TAMs and induce their M1-like phenotype instead (Zhao et al., 2021).

PI3Kγ is a molecule expressed in myeloid cells but not cancer cells, responsible for myeloid cell recruitment during inflammation and cancer. PI3Kγ inhibition demonstrated promising therapeutic potentials to induce the transition from M2 to M1-like TAMs, staggering tumor growth and survival by increasing MHC-II expression and proinflammatory cytokine secretion while decreasing the production of immunosuppressive factors in tumors and TAMs. In murine models of HNSCC, PI3Kγ inhibition synergized with anti-PD-1 therapies, contributing to greater survival and effective antitumoral activities by increasing immune response gene expressions and Teff recruitment to the TME at higher levels than PI3Kγ inhibition alone (Kaneda et al., 2016). Like CSF-1R therapies, PI3Kγ therapies are anticipated to benefit greatly from combination regimens with ICIs. IPI-549, a small molecule inhibitor of PI3Kγ, was found to increase PD-1 and CTLA-4 expressions on Teff upon administration, where additional ICI treatments are anticipated to neutralize their effects. Indeed, in ICI-resistant murine models, the combination of anti-CTLA-4 or anti-PD-1 mAbs with IPI-549 significantly delayed tumor growth in comparison to ICI monotherapies (De Henau et al., 2016). IPI-549 is actively undergoing clinical evaluation in combination with nivolumab (NCT03980041 and NCT02637531) and atezolizumab (NCT03961698) for the treatment of various cancers (Infinity Pharmaceuticals, Inc., 2021a; Infinity Pharmaceuticals, Inc., 2021b; Infinity Pharmaceuticals, Inc., 2021c).

## Cancer-Associated Fibroblasts

CAFs are defined by their ability to degrade the ECM, increase angiogenesis, and promote tumor growth and invasiveness (Mishra et al., 2011). In the TME, epithelial cancer cells release growth factors into the TME that mediate fibroblast activation, such as TGF-β, platelet-derived growth factor, hepatocyte growth factor (HGF), and epidermal growth factor. Resident fibroblasts can be activated by TGF-β binding to the ubiquitous type II serine/threonine kinase receptor TGF-βRII, where it activates TGF-βRI and SMAD. TGF-β/SMAD mediates the exosomal secretion of CXCL12/CXCR4 chemokines by CAFs, where CXCL12 promotes cancer malignancy by increasing tumor cell proliferation, migration, and angiogenesis (Kuzet and Gaggioli, 2016). HGF can induce the conversion of normal fibroblasts into CAFs and promotes tumor proliferation, migration, and ultimately cancer progression (Wu et al., 2013).

### CAF-derived Effects on Immune Cell Subtypes

CAFs affect a variety of myeloid cells, such as MDSCs, TAMs, and DCs. CXCL12 within the TME are primarily derived from CAFs (Feig et al., 2013), which recruit myeloid cells into the TME. Similarly, CXCL1 attracts PMN-MDSCs into the TME (Kumar et al., 2017). CAFs additionally secrete IL-6, inducing IDO production in DCs (Cheng et al., 2016). CXCL12/CXCR4 expression by CAFs is mediated by PGE2 and TGF-β, where the activation of the CXCL12/CXCR4 pathway helps to maintain the elevated TGF-β expression in CAFs (Kojima et al., 2010, 20; Obermajer et al., 2011). The CXCL12/CXCR4 pathway also contributes to T cell exclusion, where CXCL12 inhibitors restored the infiltration of T cells into the TME (Zboralski et al., 2017). Leukemia inhibitory factor (LIF) secreted by CAFs induces the expression of genes related to an oncogenic phenotype, such as CCL2, CCL3, CCL7, CD206, and CD163, but decreases the expression of CXCL9. While CCL2 is an essential chemokine to attract myeloid cells into the TME (Barrett and Puré, 2020), CXCL9 is an important chemoattractant for the migration of CD8^+^ T cells. LIF additionally inhibits the ability of TAMs to recruit cytotoxic T cells into the TME by using enhancer of zeste 2 polycomb repressive complex 2 subunit to silence the CXCL9 gene (Pascual-García et al., 2019). While CAFs indirectly increase the CCL2 concentration in the TME by inducing CCL2 expression in TAMs, fibroblast activation protein-positive CAFs are capable of secreting their own CCL2s by activating the fibroblastic STAT3 through a JAK2 signaling pathway (Figure 1B) (Yang et al., 2016).

The depletion of alpha-smooth muscle actin (α-SMA)^+^ CAFs led to the acceleration of tumor growth by increasing the recruitment of Tregs into the TME, suggesting a potential positive effect by CAFs in regulating T cell activity against cancer progression (Özdemir et al., 2014). However, the role of CAFs in regulating T cell activity appears to be more complex. In esophageal tumor tissues, CD8^+^ TILs were found to be negatively correlated to CAFs, while FoxP3^+^ TILs exhibited a positive correlation with CAFs. Co-culture of cancer cells and CAFs yielded high concentrations of IL-6, where blocking the IL-6 signaling resulted in decreased tumor growth and increased accumulation of CD8^+^ TILs in tumor tissues. As IL-6 is known to inhibit the TGF-β-dependent differentiation of naive T cells into Tregs (Kimura and Kishimoto, 2010), the role of IL-6 in the TME needs to be better elucidated (Kato et al., 2018).

Specific subsets of CAFs in breast cancer were found to retain CD4^+^ CD25^+^ T cells at their surfaces through OX40L, PD-L2, and junctional adhesion molecule 2 pathways to promote their differentiation into Tregs through B7H3 (CD276), CD73, and dipeptidyl-peptidase 4. The molecules described have potential roles in anti-tumor immunity: B7H3 is an immune checkpoint molecule, CD73 is involved in the adenosine pathway (Barrett and Puré, 2020), and dipeptidyl-peptidase 4 cleaves CXCL10, a chemokine with the capacity to recruit Teffs into the TME, into an antagonist to its own receptor CXCR3 (Costa et al., 2018). Furthermore, CAFs possess the ability to cross-present antigens complexed to MHC class I molecules and are involved in the direct killing of Teffs via the expression of Fas ligand and PD-L2. The ligands engage the immune checkpoints Fas and PD-1 on the surfaces of T cells to induce their death and dysfunction (Figure 1B) (Lakins et al., 2018). The expression of PD-L1 and PD-L2 by CAFs reduces the infiltration of CD8^+^ T cells (Cho et al., 2011). CAFs can additionally increase the expression of PD-L1 on tumor cell surfaces by expressing CXCL5, which engages CXCR2 on tumor cells and upregulates PD-L1 by activating PI3K/AKT signaling (Li Z. et al., 2019). TGF-β also has multiple roles in preventing the T cell response to tumors; not only does TGF-β induce the differentiation of CAFs, which increases TGF-β1 signaling in tumors, but it also inhibits CD8^+^ T cell expansion and function. However, because TGF-β is important for tissue homeostasis, targeting its downstream targets such as NADPH oxidase 4 (NOX4) can be a safer approach to restoring T cell anti-tumor activity restricted by TGF-β. The inhibitor of NOX4 was shown to prevent CAF differentiation and convert it to a normal fibroblast-like cell (Ford et al., 2020).

### CAF-Based Therapies

Therapies targeting CAFs have largely been unsuccessful due to the difficulties presented by the cell type. Development of approaches targeted at CAFs face challenges due to the heterogeneity of CAFs and the inability of murine models to emulate the stromal reactions that take place in the human TME (Hanley and Thomas, 2020). While TGF-β inhibition in combination with anti-PD-1 therapy was found to increase CD8^+^ infiltration into tumors (Mariathasan et al., 2018), no decrease in CAF levels within the TME or CD8^+^ levels at the tumor margin was observed in CAF-rich models. Although TGF-β inhibition prevents CAF activation, it fails to reverse the CAF phenotype, suggesting its limited therapeutic potential (Ford et al., 2020).

As such, the small molecule inhibitor of NOX4 has been shown to be a promising option to overcome resistance to ICI therapies by targeting CAFs downstream of TGF-β signaling. In murine models of colorectal cancer, CAFs indeed conferred tumor cells resistance to anti-PD-1 treatments by increasing CTLA-4 expression on CD8^+^ T cells. While CTLA-4 inhibition on CAF-rich lung cancer tumors demonstrated antitumor effects, it had minimal effects on T cells or tumors with low levels of CAFs. The NOX4 inhibitor GKT137831 (setanaxib) instead neutralized the immunosuppressive activity of CAFs by downregulating functional CAF markers such as α-SMA and collagen-1. With increased CD8^+^ cell tumor infiltration, GKT137831 additionally induced the increased expression of PD-L1 by colorectal tumor cell. The combination of GKT137831 and anti-PD-1 therapy resulted in the greater tumor infiltration by CD8^+^ T cells and overall survival relative to anti-PD-1 therapy alone. Surprisingly, the depletion of TAMs using anti-CSF-1R inhibitors on CAF-rich tumors had minimal effects, suggesting the importance of characterizing the immune microenvironment to identify their primary immunosuppressive cell populations (Ford et al., 2020). GKT137831 has yet to enter clinical trials as monotherapy or in combination with ICIs.

Instead of turning off the signals necessary for CAF maintenance, a different strategy is to activate signals that maintain the “normal” fibroblast phenotype. In CRC, high vitamin D receptor (VDR) expression by CAFs was associated with longer CRC patient survival (Ferrer-Mayorga et al., 2017). VDR functions as a master transcriptional regulator, where its activation suppresses tumor-supporting signaling pathways (Sherman et al., 2014). The most active vitamin D metabolite, 1α,25-dihydroxyvitamin D_3_ (1,25(OH)_2_D_3_), reduced the expression of activated fibroblast marker *S100A4* and the ability of CAFs to induce CRC cell migration. This effect was observed across fibroblasts not only from CRC patients but from human lung, foreskin, and mouse embryo tissues (Ferrer-Mayorga et al., 2017). In the context of pancreatic cancer, pancreatic stellate cells (PSCs), the predominant fibroblast in the TME of the pancreas, have impaired tumor-promoting capacities in response to vitamin D receptor (VDR) engagement VDR signaling was shown to support signaling pathways to promote a quiescent state of PSCs and increase chemotherapy efficacy (Sherman et al., 2014). The VDR agonist paricalcitol is currently in phase I and II clinical trials in combination therapy with the chemotherapeutic agent gemcitabine to evaluate its safety and efficacy in treating pancreatic cancer (NCT03520790 (Perez, 2021) and NCT04617067 (Cancer Trials Ireland, 2021)) Table 1.

Despite these potential therapeutic benefits of VDR, Gorchs et al. recently discovered the conflicting roles of VDR agonists in pancreatic cancer therapies. Calcipotriol, a vitamin D_3_ analogue, was shown to increase the expression of α-SMA but reduce the secretion of IL-6 and LIF. Additionally, calcipotriol decreased the CAFs’ proliferative and migratory capacities, potentially reversing their immunosuppressive phenotypes. Surprisingly, calcipotriol significantly reduced CD8^+^ T cell function and proliferation, primarily by vitamin D’s ability to promote tolerogenic DCs and produce Tregs (Xie et al., 2017; Gorchs et al., 2020). However, calcipotriol increased the expression of PD-L1 on CAFs but decreased their PD-L2 expression, creating an opportunity for anti-PD-1 therapies to circumvent the pro-tumoral effects. Preclinical studies on VDR agonist treatments face further challenges due to the difference in VDR signaling between murine and human biology (Gorchs et al., 2020). Further clinical evaluation is therefore required to validate the efficacy of VDR agonists in affecting human CAF activities.

Another signaling pathway of interest has been the CXCL12/CXCR4 pathway, which recruits immunosuppressive cells while excluding Teffs. NOX-A12 is an RNA oligonucleotide drug that binds CXCL12 with high affinity and effectively inhibits the interactions with its ligands, CXCR4 and CXCR7. In preclinical studies, NOX-A12 synergized with anti-PD-1/PD-L1 therapy by enhancing Teff infiltration into the tumor (Zboralski et al., 2017). Clinical trials of NOX-A12 in combination with pembrolizumab for the treatment of microsatellite-stable CRC or pancreatic cancer demonstrated excellent overall tolerability and efficacy even in heavily pretreated patients who underwent multiple lines of chemotherapy. Responses were attributable to the increased CD3^+^ T cell infiltration and interferon production. However, the authors did not biopsy tumor samples that received the combination treatment, leaving the post-treatment characterization of the TME left much to be desired (Suarez-Carmona et al., 2021). Currently, NOX-A12 is undergoing additional phase II clinical trials in combination with pembrolizumab to evaluate both its safety and toxicity of the therapy in the context of microsatellite-stable metastatic pancreatic cancer (NCT04901741 (NOXXON Pharma AG, 2021)) Table 1. Identifying the synergistic mechanisms between CXCL12 inhibitors and ICIs will help determine the most effective treatment regimens.

## Tumor Hypoxia

Hypoxia provides tumor growth advantage by exercising several immune suppressive mechanisms (Hompland et al., 2021). It is well established that hypoxic conditions create increased mutational burden in tumor cells leading to heterogeneity and eventual immune escape (Noman et al., 2019; Terry et al., 2020). Tumor hypoxia shields cancer cells from immune surveillance by modulating various regulatory pathways. An immediate outcome of hypoxia is the upregulation and stabilization of the transcription factor HIF. Of the different hypoxia inducible factors, HIF-1α plays an integral role in conferring resistance to immune cell attack by transcriptional regulation of key survival genes in tumors.

Under hypoxic conditions, immune-suppressive actions can additionally be triggered by over-expression of VEGF and activation of VEGF receptor. The master regulator HIF-1α activates immune-suppressive effects by recruiting and stimulating immune-suppressor cells (Treg, MDSC), inducing secretion of immune-suppressive Th2-type cytokines, and inhibiting antitumor immune responses. The latter inhibitory effect is carried out mainly by suppressing the effects of immune cells such as NK, natural killer T (NKT), CD4^+^ and CD8^+^ T cells, curtailing antigen-presenting DC cells, and reducing immune-stimulatory Th1-type cytokines (Vaupel and Multhoff, 2018).

Cancer cells adapt to low oxygen levels of hypoxia by metabolic shift, deriving their energy by converting glucose to lactate rather than by aerobic glycolysis/TCA cycle. Even though energy yield in glycolysis is much lower in glycolysis compared to TCA cycle (2 ATPs compared to 36 ATPs), tumor cells utilize the available resources for catabolic processes. In this way, they take the advantage of the situation by producing more biomass for sustainability rather than just increased energy production. Due to increased aerobic glycolysis, lactic acid is generated and released into the TME creating an acidic milieu that is inhospitable for immune cells (Lardner, 2001; Brand et al., 2016; Lim et al., 2020). Tumor acidity could also have a profound effect on the bioactivity and distribution of antibodies, thus potentially dampening the clinical efficacy of therapeutic antibodies (Huber et al., 2017).

However, the effector functions of immune cells that are inhibited by lactic acid and an acidic TME have been experimentally demonstrated to be reversible in a variety of immune cell types across different cancers (Calcinotto et al., 2012). Thus, if the acidic TME can be buffered back to a physiological condition, the anticancer functions of various immune cells can likely be restored—uncovering a potential for an extremely powerful form of immunotherapy. In a preclinical study, Pilon-Thomas et al. examined the effect of pH buffering in the context of cancer immunotherapy (Pilon-Thomas et al., 2016). Bicarbonate administration was added on to the treatment regimen involving anti-PD-1 antibodies, where the combination showed improved antitumor response in different tumor types to indicate that reversing tumor acidity could be a better treatment option in immune checkpoint blockade therapies. Though the exact mechanism was not apparent, it was evident that more T cell homing to the tumor site could have a played role in tumor suppression. In another study reported by Chafe et al., carbonic anhydrase IX inhibition by SLC-0111 showed decreased TME acidification in part due to reduced glycolytic metabolism of tumor cells, which in turn increased immune activity (Chafe et al., 2019).

### Hypoxia-Targeting Therapies

Among the biomarkers for predicting the outcome of immunotherapy, the hypoxic milieu is often overlooked even though it is the basic niche from which complications arise. The extent of hypoxia in a tumor could be an important biomarker to estimate immunotherapy outcomes (Wang B. et al., 2021). A hypoxia-immune based gene signature was constructed by Yang et al. in triple negative breast cancer as a predictive model for risk stratification and survival (Yang et al., 2021). Their model was derived from the existing data in different databases, identifying six cross-cohort prognostic hypoxia-immune related gene signature. The robustness of this model was also validated among different groups of triple negative breast cancer patients, highlighting the importance of hypoxic TME when considering immunotherapy. Furthermore, the negative impact of hypoxia on the tumor immune response by modifying the expression of main immune checkpoints could be advantageous for developing innovative combination approaches (Noman et al., 2019) Table 1. As such, it has been suggested that targeting the hypoxic TME would enhance immunotherapy to a great extent (Abou Khouzam et al., 2020). Several immunotherapy studies targeting the hypoxic TME have also been carried out under preclinical and clinical settings, which include the use of hypoxia-activated prodrugs and the inhibition of HIF signaling (Terry et al., 2018).

Hypoxia activated prodrugs are drugs that undergo bioreduction in low-oxygen conditions to yield cytotoxic metabolites. Currently, several hypoxia activated prodrugs are available, where TH-302 (evofosfamide) is widely used as a combinatorial agent in immunotherapy (Guise et al., 2014; Phillips, 2016; Noman et al., 2019). Under hypoxic conditions, TH-302 is reported to reduce the expression of HIF-1α, induce cytotoxicity by DNA crosslinking, and inhibit cell proliferation (Meng et al., 2012). In an *in vivo* mouse study, TH-302 in combination with PD-1 and CTLA-4 inhibitors significantly suppressed prostate cancer and extended the survival period. In this study, immunotherapy alone was not efficient as the hypoxic regions in prostate cancer models lacked T cell infiltration, creating immunotherapy resistance zones. Adding TH-302 to the therapeutic regimen resulted in the suppression of MDSCs while also increasing the recruitment of T cells into hypoxic tumor regions (Jayaprakash et al., 2018). TH-302 also showed favorable results in controlling soft tissue sarcoma when used in combination with Adriamycin (Phillips et al., 2013). TH-302 also showed favorable results in controlling soft tissue sarcoma when used in combination with Adriamycin (Phillips et al., 2013), and similarly, Jamieson et al. reported that the combined therapy of TH-302 and CTLA-4 blockade improved the survival rate in a HNSCC model compared to the use of a single agent alone (Jamieson et al., 2018). In a recent clinical trial, TH-302 is included in the combination therapy with ipilimumab and Adriamycin, where in the latter case, it is used against cancer models of pancreatic, prostate, and melanoma. The results of this ongoing clinical trial (NCT03098160 (Threshold Pharmaceuticals, 2017)) are much awaited to evaluate its performance. However, as promising as they are in overcoming therapy resistance in tumors, hypoxia activated prodrugs have not always been successful in the clinic. The main reason for suggested for this shortcoming is the lack of patient stratification based on tumor hypoxia status, which is highly variable among patients. Thus, the stratification of patients is an essential factor to achieve successful therapy with the hypoxia activated prodrugs (Spiegelberg et al., 2019).

Inhibition of HIF signaling is also one of the avenues to enhance immunotherapy in several cancers exhibiting immunotherapy resistance. Developing pharmacological agents to modulate HIF-1α signaling has inspired significant interest in recent times. Several drug sub-types have been described to inhibit HIF-1α activity and include inhibitors of HIF-1α/HIF-1β dimerization (e.g. acriflavine), HIF-1α degradation (e.g.Bisphenol A), HIF-1α protein synthesis and stability (e.g. Glyceollins) (Semenza, 2012; Ellinghaus et al., 2013; Scholz and Taylor, 2013). In a technique mimicking the mode of activation of acriflavine, HIF-1α transcriptional activity was compromised by deleting the domain essential for dimerization with HIF-1β, leading to successful inhibition of melanoma (Lequeux et al., 2021). In RCC, adding HIF inhibitors has boosted the immunotherapy outcome in therapy resistant patients. Collective data now indicates that combinations of immune checkpoint inhibitors, HIF signaling inhibitors, and cytokines are powerful regimens to address this cancer type (Considine and Hurwitz, 2019). In a phase I clinical trial, another HIF-2α inhibitor, PT2385, in combination with anti-PD1 mAb exerted a higher synergistic inhibitory effect on clear cell RCC in comparison with single agent treatment alone (Ban et al., 2021).

In recent times, hyperbaric Oxygen therapy (HBO) is also gaining momentum as a treatment modality in some of the cancer types with elevated HIFs. HBO mainly targets the HIF axis to suppress cancers, although additional primary or secondary targets of the therapy cannot be ruled out (Zhang et al., 2021). Most recently, HBO therapy has been combined with immunotherapy in a phase I trial (2021–2024) to treat cancer patients with resistance to previous immunotherapy (NCT05031949 (bixiangzhang, 2021)). The study will explore the efficacy and safety of HBO therapy plus camrelizumab as a second-line treatment.

## Exosomes

Exosomes consist of EVs containing molecules such as lipids, proteins, short RNAs, long noncoding RNAs (lncRNAs), and microRNAs (miRNAs) (D’Asti et al., 2016). Exosomes produced by cancer cells are classified as tumor-derived exosomes (TEX), responsible for facilitating immunosuppression by mediating the development, maturation, and function of immune cells within the TME (Whiteside, 2016). Besides affecting immune cells, tumor cells can secrete TEX to induce angiogenesis or self-proliferation for their own maintenance, or promote the transformation of normal cells into cancer or cancer-promoting cells (Skog et al., 2008). Exosomes from stromal cells, such as fibroblasts, were additionally shown to support tumor growth by providing nutrients, inhibiting apoptosis of tumor cells, and driving their proliferation (Gurunathan et al., 2019).

### LncRNAs

The delivery of lncRNAs via exosomes can promote tumor progression. LncRNA from TEX promotes angiogenesis by stimulating circulating angiogenic cells. The lncRNA HOX antisense intergenic RNA (HOTAIR) contained in TEX from glioma cells affects endothelial cells by promoting their expressions of VEGF-A (Sun et al., 2018). In bladder cancer, HOTAIR promotes the invasion of urothelial bladder cancer cells by upregulating the genes associated with EMT, such as the Snail family transcriptional repressor 1, Laminin subunit gamma 2, and laminin subunit beta 3 (Berrondo et al., 2016). In the context of CRC, the expression level of the exosomal lncRNA 91H was found to be positively associated with the risk of tumor relapse or metastasis in a heterogeneous nuclear ribonucleoprotein K-dependent mechanism (Gao et al., 2018). Similarly, the lncRNA H19 promotes HCC growth, angiogenesis, and metastasis by upregulating VEGF in endothelial cells and promoting the adhesion of CSC-like liver cells to endothelial cells (Matouk et al., 2007; Conigliaro et al., 2015; Niu et al., 2017).

### miRNAs

Another type of RNA that can be carried within exosomes are microRNAs. Tregs secrete exosomes containing miR-150-5p and miR-142-3p to cause DC dysfunction in addition to delivering exosomal miR-let-7d to Th1 cells, crippling their effector functions. Tumor cells create an immunosuppressive TME by secreting exosomes carrying miRNAs such as miR-21 to induce M2-like polarization in monocytes, while exosomal miR-21 promotes the activation of MDSCs to drive immunosuppression within the TME (Tan et al., 2020). miR-122 is another miRNA-containing TEX secreted by breast cancer cells, reducing glucose uptake in normal, noncancerous cells by downregulating their expression of pyruvate kinase. Reduction of glucose consumption by other cells promotes cancer growth by increasing glucose availability to tumors, and supports cancer metastasis by influencing pre-metastatic niches to facilitate tumor cell colonization and metastatic formation (Fong et al., 2015).

TEXs carrying miRNAs and circular RNAs (circRNA) can serve as mediums facilitating tumor immune escape and conferring tumor cells resistance to immunotherapy. In the context of glioma, hypoxic glioma-derived exosomes were found to carry miR-1246, which induces M2 macrophage polarization in the TME to drive tumor progression and survival. MiR-1246 targets the telomeric repeat binding factor 2 interacting protein, which subsequently activates STAT3 and inhibits the NF-kB signaling pathway in macrophages, ultimately contributing to immunosuppression (Qian et al., 2020). Furthermore, endoplasmic reticulum stress on HCC cells were found to release exosomes containing miR-23a-3p to TAMs. MiR-23a-3p inhibited the TAM’s phosphatase and tensin homolog expression, activating the PI3K-AKT pathway to upregulate their PD-L1 expression (Pascut et al., 2020). Ubiquitin-like with PHD and RING finger domain 1 (UHRF1) is a molecule usually overexpressed in cancer, which is normally responsible for the regulation of DNA methylation. In the context of HCC, the overexpression of UHRF1 promotes tumorigenesis and cancer progression. Not only is the UHRF1-derived circRNA (circUHRF1) closely correlated to poor HCC prognosis, but the HCC-derived exosomal circUHRF1 also degrades the miR-449c-5p in NK cells to upregulate their TIM3 expressions. CircUHRF1 is also implicated to confer resistance to anti-PD-1/PD-L1 therapy, as demonstrated by the increased sensitivity of circUHRF-1-knockdown HCC cells to anti-PD-1/PD-L1 treatment (Zhang P.-F. et al., 2020).

### Proteins

Besides RNA, exosomes can contain proteins to drive resistance to immunotherapies (Sun et al., 2018). For example, tumor-derived exosomal PD-L1 has been implicated for its role in driving resistance to anti-PD-1/PD-L1 therapy. Tumor cells secrete exosomes containing PD-L1 both on its surface and inside the exosome, upon which can be transferred to neighboring cells without PD-L1 expression and bind to PD-1 to impair T cell activation (Yang et al., 2018). The expression of PD-L1 on these vesicles is upregulated by IFN-γ (Chen et al., 2018). Exosomal PD-L1 display greater immunosuppressive effects than soluble PD-L1, primarily due to the effect of exosomal MHC-I interaction with TCR enhancing the inhibitory effect of exosomal PD-L1. Like PD-L1 on tumor cells, exosomal PD-L1 unleashes devastating immunosuppressive effects on T cells, promoting the apoptosis of CD8^+^ T cells, suppressing their proliferation and effector functions, and instead driving the inhibitory activities of Tregs (Yin et al., 2021). The pre-treatment levels of circulating exosomal PD-L1 was significantly higher for melanoma patients who did not respond to pembrolizumab. However, if anti-PD-1/PD-L1 therapy blocks the interaction between PD-1 and PD-L1, how would exosomal PD-L1 confer resistance if it is blocked in the same way? One possible explanation for this phenomenon is that high pre-treatment levels of exosomal PD-L1 may have driven T cells to a point of exhaustion beyond rescue by anti-PD-1/PD-L1 therapy (Chen et al., 2018). Another speculated mechanism is that the delivered anti-PD-L1 mAbs may not be sufficient to block both the exosomal and surface-expressed PD-L1, ultimately leading to T cell inhibition. However, the exact mechanism of exosomal PD-L1-derived resistance still remains elusive, and therapeutic approaches to deplete exosomal PD-L1 from the TME can be investigated, as its removal led to enhanced responses to anti-PD-1/PD-L1 treatments (Yin et al., 2021).

### Exosome-based Therapies

Exosomes can be utilized as immunotherapy mediums to deliver appropriate stimulatory signals and carry out effective antitumor immune responses. For example, DC-derived exosomes (DCexos), which contain MHC I, MHC II, and CD86 can activate CD4^+^ and CD8^+^ T cells (Viaud et al., 2010). DCexos loaded with melanoma antigen gene tumor antigens were found to induce melanoma antigen gene-specific T cell responses and increase NK cell lytic activities (Morse et al., 2005). While DCexos, as inert vehicles, exhibit resistance to tumor-derived suppressive factors in addition to their greater bioavailability and biostability, they fail to induce appropriate levels of T cell activation and suffer from low response rates (Fu C. et al., 2020). Instead, synthetic exosomes (iExosomes) can be engineered to incorporate a variety of agents to induce antitumor responses. iExosomes loaded with oxaliplatin (OXA), a chemotherapeutic agent for the treatment of pancreatic ductal adenocarcinoma, and gal-9 small interfering RNA (siRNA) were delivered to the tumor sites of murine pancreatic cancer models. The respective exosomes displayed specific pancreatic tumor-targeting abilities and the downregulation of gal-9 by pancreatic tumor cells to ultimately induce the M1-like polarization of local TAMs. While exosomes loaded with OXA or gal-9 siRNA were unsuccessful in restraining tumor growth alone, their combination regimen significantly decreased tumor size and prolonged survival relative to the effects of the conventional chemotherapeutic agent gemcitabine (Zhou et al., 2021).

Another promising target is the KRAS oncogene, which is a primary drug target in lung cancer. Mutation in KRAS lock it in an active state, sending downstream signals that cause cancer by increasing cell proliferation and survival (Stephen et al., 2014). However, inhibitors targeting KRAS directly faces challenges due to the absence of deep hydrophobic pockets available for binding on the KRAS molecule itself (Cox et al., 2014). As such, the delivery of EFTX-D1, a siRNA selectively targeting the most common mutated KRAS genes such as G12C, G12D, and G13D, has been explored as a therapeutic approach. EFTX-D1 was able to decrease both the levels of oncogenic KRAS mRNA and protein levels, but *in vivo* preclinical trials are yet to be conducted (Papke et al., 2021). In agreement with Papke et al., the authors agree that the nanoparticle delivery of EFTX-D1 using iExosomes will be the next step to evaluate the KRAS inhibitor’s performance, possibly in combination with ICIs. The ongoing clinical trial delivering KRAS G12D siRNA in mesenchymal stromal cell-derived iExosomes to pancreatic cancer patients with KRAS G12D mutations will be beneficial to elucidate the challenges and therapeutic efficacy of delivering KRAS siRNAs with iExosomes (NCT03608631 (M.D. Anderson Cancer Center, 2021b)) Table 1.

A different approach is to target exosomes using GW4869, which prevents the secretion of exosomes by inhibiting neutral sphingomyelinases (Menck et al., 2017). In the context of breast cancer cells, the administration of GW4869 ameliorated the metabolic changes in the TME caused by CAF-derived exosomes in preclinical trials. CAF-derived exosomes contributed to the immunosuppressive TME by decreasing the oxygen consumption rate by tumor cells and increasing lactate levels, where GW4869 administration partially negated this metabolic change (Li Y. et al., 2020, 3). However, the off-target effects of GW4869 administration needs to be assessed in detail, or ways to specifically target certain CAF-derived exosomes are required for maximal benefits.

## Concluding Remarks

Without a doubt, ICI immunotherapy revolutionized the landscape of cancer treatments in the last decade. While immunotherapy exhibits remarkable potential, there are numerous obstacles within the TME that make it difficult to achieve high response rates and sustained benefits for patients. Immunosuppressive cells, hypoxic conditions, metabolites, and exosomes are among the many factors that contribute to immunotherapy resistance.

Today, there are numerous clinical trials being conducted investigating the different combinations of immunotherapies, which exhibit greater response rate and efficacy. It is evident that targeting multiple inhibitory pathways in the TME contributing to immune escape is a promising approach to enhance anti-tumor activities by immune cells and staunch tumor progression. Incorporating other ICIs beyond anti-CTLA-4 or anti-PD-1/PD-L1 to be used in combination therapies will be the next step in overcoming resistance to immunotherapies involving ICIs, offering patients additional options. Additionally, disabling or clearing the TME of immunosuppressive populations or compounds responsible for ICI resistance will allow T cells to exert their antitumoral effects. Broadening our knowledge of the TME will ultimately bring us closer to the goals of increasing both the number of patients eligible for ICI therapies and the response rates to treatments.
